# Bryo-Activities: A Review on How Bryophytes Are Contributing to the Arsenal of Natural Bioactive Compounds against Fungi

**DOI:** 10.3390/plants10020203

**Published:** 2021-01-21

**Authors:** Mauro Commisso, Francesco Guarino, Laura Marchi, Antonella Muto, Amalia Piro, Francesca Degola

**Affiliations:** 1 Department of Biotechnology, University of Verona, Cà Vignal 1, Strada Le Grazie 15, 37134 Verona (VR), Italy; mauro.commisso@univr.it; 2 Department of Chemistry and Biology, University of Salerno, Via Giovanni Paolo II 132, 84084 Fisciano (SA), Italy; fguarino@unisa.it; 3 Department of Medicine and Surgery, Respiratory Disease and Lung Function Unit, University of Parma, Via Gramsci 14, 43125 Parma (PR), Italy; laura.marchi@unipr.it; 4 Department of Biology, Ecology and Earth Sciences, University of Calabria, Via Ponte P. Bucci 6b, Arcavacata di Rende, 87036 Cosenza (CS), Italy; antonella.muto@unical.it; 5 Laboratory of Plant Biology and Plant Proteomics (Lab.Bio.Pro.Ve), Department of Chemistry and Chemical Technologies, University of Calabria, Ponte P. Bucci 12 C, Arcavacata di Rende, 87036 Cosenza (CS), Italy; amalia.piro@unical.it; 6 Department of Chemistry, Life Sciences and Environmental Sustainability, University of Parma, Parco delle Scienze 11/A, 43124 Parma (PR), Italy

**Keywords:** bryophytes, plants bioactive compounds from early-diverged land, natural antifungals, plant extracts, mosses, liverworts, hornworts

## Abstract

Usually regarded as less evolved than their more recently diverged vascular sisters, which currently dominate vegetation landscape, bryophytes seem having nothing to envy to the defensive arsenal of other plants, since they had acquired a suite of chemical traits that allowed them to adapt and persist on land. In fact, these closest modern relatives of the ancestors to the earliest terrestrial plants proved to be marvelous chemists, as they traditionally were a popular remedy among tribal people all over the world, that exploit their pharmacological properties to cure the most different diseases. The phytochemistry of bryophytes exhibits a stunning assortment of biologically active compounds such as lipids, proteins, steroids, organic acids, alcohols, aliphatic and aromatic compounds, polyphenols, terpenoids, acetogenins and phenylquinones, thus it is not surprising that substances obtained from various species belonging to such ancestral plants are widely employed as antitumor, antipyretic, insecticidal and antimicrobial. This review explores in particular the antifungal potential of the three Bryophyta divisions—mosses (Musci), hornworts (Anthocerotae) and liverworts (Hepaticae)—to be used as a sources of interesting bioactive constituents for both pharmaceutical and agricultural areas, providing an updated overview of the latest relevant insights.

## 1. Introduction

Today as in the earliest terrestrial environment, plants are frequently found to manage their growth under adverse situations in terms of both abiotic stress and biotic interactions [1]: unfavorable conditions including temperature and UV radiation injuries, unpredictable water availability, predation and pathogens attack triggered—and still represent—an evolutionary pressure that challenges the plasticity of plants defense arsenal. The exploitation of their chemical weapons for pharmacological purposes dates back to several decades in a number of fields of application, relying on the observation that plant metabolites successfully evolved/selected for antagonistic activities against microbial pathogens could be employed as the bioactives reservoir, suitable in their native form or being specifically modified to tailoring their biological effect. So far, turning to compounds produced by a plant species to fight microorganisms belonging (and adapted) to its ecological niche has been a winning strategy, as countless are the plant-derived molecules actually present on the drug market in the form of pharmacophores, biocides and other antimicrobial agents. However, due to the incessant struggle between organisms for survival, an increasing occurrence of resistance phenomena is arising, making the race towards new and effective bioactive compounds more cogent. At the same time, the documented adverse effects on the environment and human health, the loss of efficacy by the most commonly commercialized synthetic pesticides, fungicides and insecticides, due to an indiscriminate use, has increased the need to provide new natural products as an alternative to hazardous chemicals. 

Plants stand as an infinite resource for drug development: it has been estimated that the 11% of the 252 drugs considered as basic and essential by the WHO are obtained from flowering plants [2] but a wide amount of phytochemicals provided with antimicrobial properties was characterized and isolated in representatives of each taxa, from algae to pteridophytes to higher plants; bryophytes, non-vascular early land plants considered the link between seed and vascular plants to their algal ancestors, also contribute to this “phytochemical treasure chest”. Probably, because the necessity of defense against detrimental microorganisms has characterized the land life since the very beginning, and the lack of mechanical protection typical of the more equipped vascular plants was counterbalanced by the biochemical capability in devising active molecular architectures as a part of the survival strategy.

Bryophytes can be found in every type of habitat, except in the oceans, thus reflecting the positive species expansion occurred along the evolution. However, bryophytes rarely prevail in the ecosystems, probably due to the absence of special structures that prevent water loss (such as thick cuticles, epidermis and functional stomata), the lack of a highly specialized system for water transport and distribution inside the plant, and a water-dependent fecundation process.

More than a few biochemical adaptations provided the essential requirements for colonization and diversification on land: for example, the ability to establish close interactions with bacteria and fungi is thought a critical innovation to ensure access to limited and scattered nutrients, as revealed by the observation that arbuscular mycorrhizal fungi were found to regularly colonize not only flowering plants but also liverworts and hornworts [3]. Unprovided with mechanical protections typical of cormophytes, shielded by cuticle or bark against microbial pathogens, Bryophytes survived for more than 350 million years due to their alternative poikilohydric life strategy, taking advantage of chemical weapons: for example, the release of phenolic compounds from wetted thalli and phylloids is intended, and strongly effective, to inhibit the germination of fungal spores that occasionally land on their surface. Thus, inevitably induced to biosynthesize secondary metabolites functional to limit biotic stressors, bryophytes are relatively unaffected by microbial diseases although usually growing in humid habitats, being able to elaborate constitutive or inducible small-molecule with antimicrobial properties [4]. Therefore, they represent a valuable source of interesting bioactive compounds: a number of terpenoids (the largest group of secondary metabolites from bryophytes with more than 1600 structures described) [5] and aromatic compounds (mainly flavonoids, phenylpropanoids, benzenoids and bibenzyl derivatives) have been isolated [6,7,8]. Some of them showed an interesting antimicrobial potential: several species of liverworts, including *Plagiochila*, *Bazzania* and *Radula* species, *Conocephalum conicum*, *Dumortiera hirsuta*, *Marchantia polymorpha*, *Riccia gangetica, Metzgeria furcata, Lunularia cruciata* and *P. vernicosa* complex, proved to contain metabolites, such as marchantin A and lunularin, that displays strong antifungal activity against various *Aspergillus*, *Penicillium*, *Fusarium*, *Candida* and *Rhizoctonia* spp. [9,10,11]. Others, *Trichocolea mollissima, T. tomentella* and *T. lanata*, possess prenyl-phenyl ethers that are mildly fungicidal [12,13]. Recently, a structural study aimed at understanding the structure of early land plants through the comparison of the tube and cell-sheet from Cambrian-to-Devonian microfossils with experimentally degraded modern liverworts, suggested that the presence of phenolic compounds was responsible for the protection of lower epidermal tissues from the soil microbe attack, and provided dimensional stability to the remains of early marchantioid liverworts similar in some ways to modern *Marchantia* and *Conocephalum* [14]. The class of bis(bibenzyl)s, characteristic constituents of liverworts, was proposed to be the main responsible for their antimicrobial effects; in particular, a significant antifungal activity was reported against the fluconazole-sensitive and resistant strains of *Candida albicans*, where the administration of some bis(bibenzyl)s also facilitated the accumulation of fluconazole in fungal cells when used in combination [15,16,17,18].

The efficacy of organic extracts against different fungal genera was reported for various mosses, at different extents: interesting inhibitory effects were reported for *Syntrichia ruralis*, *Grimmia anodon* and *Pleurochaete squorrosa* on *S. cerevisiae*, while *C. albicans*, which resulted in being insensitive to extracts obtained from these species, was strongly affected by the *Tortella tortuosa* acetone extract [19]. Aqueous crude extracts of *Bryum argenteum* and *B. cellulare*, cosmopolitan mosses, have been demonstrated to display a containment activity, at varying degrees, against the two phytopathogenic fungal species *Curvularia lunata*, the etiological cause of leaf spot of wheat, and *Drechslera maydis*, responsible for leaf blight in *Zea mays* [20,21]. The inhibitory effect was described not only in terms of hyphae elongation, but also in terms of spore germination [22], as previously reported for *Philonotis revoluta* against the fungus *Helminthosporium turcicum*, another etiological agent of “corn leaf blight” [11]. Other phytopathogenic fungi were found to be susceptible to crude extracts from moss species: *Aspergillus niger, Fusarium moniliforme* and *Rhizoctonia bataticola* development resulted in being highly impaired by the exposure to *Thuidium delicatulum* and *T. cymbifolium* extracts [23], hence highlighting the potential of such Bryopsida as a source of mycoherbicides to control plant and crop pathogens.

## 2. The (un)Covered Association

Bryophytes are non-vascular land plants exhibiting a peculiar haplodiplontic life cycle in which the gametophytic generation is dominant. Bryophytes macrogroup is composed of three different divisions: the hornworts (Anthocerophyta), liverworts (Marchantiophyta) and mosses (Bryophyta). The estimated number of bryophyte species is over 24,000 [24], making this macrogroup the second largest group of terrestrial plants, second only to the most recently evolved flowering plants. A recent classification described that 250, 7000 and 12,000 bryophyte species belong to the hornworts, liverworts and mosses divisions, respectively [25].

The long time accepted idea that early land plants were not interested by fungal association has been almost overwhelmed by the copious findings that over the years showed the contrary. Despite the persistence of such an erroneous misconception—sometimes attributed to the lack of collaboration between bryologists and mycologists, and to sampling bias for healthy specimens—fungal species forming associations with taxonomically various mosses have been reported since 1951, when Wilson individuated in some basidiomycetous pathogens the real cause of “fairy rings” in Antarctic moss prairies [26] and continued through the following decades [27,28,29,30,31]. As masterfully reviewed by Davey and Currah [32], various types of association, both pathogenic and beneficial, actually occur in the “mosses chamber” of the bryophyte’s world. In terms of their development, these interactions clearly differ from those occurring between fungi and higher plants, since characteristic targets for predation (such as storage organs and specialized nutrient transport tissues) and entrance (regulated stomata and lignified, reinforced cell walls around vessels) are likely missing in bryophytes: thus, the exploitation of degradative enzymes secreted from the penetration peg seems to represent the most common entry-ticket strategy, for example in *Sphagnum* pathogenic fungi [33,34,35]. The functional relationship with fungal symbionts, historically conceived as a distinctive trait of land plants, has been reviewed in 2010 by Pressel and colleagues, which investigated the phylogenetic implications of bryophyte–fungal associations unraveling the early evolution of fungal symbioses at the base of the land plant tree [36]: through the combination of both cytological and sequencing available data, a more than surprising endophytes diversity was noteworthily found as consistent with phylogenies. Despite several leafy liverworts families (Jungermanniideae), around 30% of liverwort species worldwide, are reportedly presenting ascomycete rhizoidal symbionts (while lesser than 100 species with basidiomycetes, glomeromycetes and mucoromycetes) [37], a demonstration of nutritional mutualisms have been achieved in a few of early-diverging thalloid and Haplomitriopsida liverworts only and, very recently, in the leafy *Cephalozia bicuspidata* colonized by the ascomycete fungus *Pezoloma ericae* [38]; the latter in particular was then established as a mycorrhiza-like symbiosis, sanctioning the Ascomycota as mycorrhizal fungal groups engaging in mutualisms with plants across the land plant phylogeny.

However, bryophyte-associated fungi must share with vascular plants-associated cousins the exigency to cope with the host defenses, evolving structural and functional adaptation to their ecological niche and host habit; questions like: how do bryopathogenic species overcome the occurrence of prolonged desiccation of their host? or what is the actual mechanism of host cells disruption exerted by these pathogens, when, in most cases, the absence of penetrating structures has been noticed? are still to be fully addressed. On the other hand, in a few species of mosses, some pathogen-response tools—such as resistance homologous genes and an oxidative burst triggered by hydrogen peroxidases activity during the fungal spread—have been found in common with higher plants [39,40] and, recently, similar but more detailed findings at both the transcriptional and proteomic level have been reported for the liverwort *Marchantia polymorpha*: Carella and coauthors, in fact, showed in 2019 that in *M. polymorpha* the infection by the phytopathogenic oomycete *Phytophthora palmivora* activates a phenylpropanoid metabolic pathway, displaying same deterrence strategy that, million years later, will evolve in the phenyl-propanoids-associated biochemical defenses so well characterized in Angiosperms [41]. Hence, it could be easily speculated that these compounds (namely polyphenols, flavonoids and anthocyanins), providing an ancestral layer of biochemical defenses in mitigating pathogen infection in liverworts, played a critical role for the terrestrialization of plants.

## 3. Phytochemistry of Bryophyte’s Antifungal Metabolites

Bryophytes evolved a vast chemical arsenal with more than 3000 different metabolites [42,43,44]; in particular, the most representative class with 2200 different molecules are terpenoids, followed by phenolic compounds (several hundred) and other molecules, such as saccharides, lipids, nitrogen- and sulfur-containing compounds [25]. During decades, as dedicated investigations have been carried out, the enumeration of antifungal compounds detected in bryophytes is continuously increasing. Biological activities were both reported from crude plant extracts in various solvents and purified compounds; in the first case, as diffusely described for higher plants, the effectiveness of a bryophyte extract in exerting the fungicide/fungistatic effect strongly depends on its balsamic period, and on seasonal, territorial (latitude and altitude and substrate composition) and climate variations that specifically influence the phytochemical composition of the plant tissues and, in turn, determine their pharmacological potential [45].

Aside from abiotic factors, particular, even poorly investigated, biotic interactions have been revealed to be connected with the antifungal potential of bryophytes, as in the case of the association between bacteria and *Tortula ruralis*, *Aulacomnium palustre* and *Sphagnum rubellum*, typical moss species belonging to nutrient-poor plant communities. Through the analysis of the antagonistic activity of bacterial isolates against the phytopathogenic fungus *Verticillium dahliae,* a high percentage (99%) of moss-associated bacteria was found to produce antifungal compounds [46], suggesting genus *Sphagnum* as a promising source of antagonistic molecules against human fungal pathogens [47]. Although their acknowledged antimicrobial potential, the bryophytes have not been fully characterized chemically and pharmaceutically, also due to the difficulty of classification and collection of a significant number of species; however, to date, the various attempts to fill this gap have successfully determined notable progress, shedding light on a plethora of metabolites with acknowledged, or potentially even not assessed yet, antifungal properties. As follows, the major secondary metabolites of these early-diverged land plants, grouped depending on their chemical features, are reported and discussed.

### 3.1. Terpenes 

Plant-derived bioactives are mostly labeled as an antioxidant: these phytochemicals are redox-active molecules, and, dynamically involved in maintaining the redox balance in the cell, have been frequently proven to exert an inhibitory effect on the fungal growth and development. Terpenoids, flavonoids and bibenzyls are the most widely described antioxidants found in the secondary metabolism of bryophytes [42], showing a high level of variability, in terms of relative abundance, within different species; for example, amongst liverworts, the chemical profile of *Radula* species is very distinctive with respect to other species: in fact, if they mainly elaborate bibenzyls (including bibenzyl cannabinoids and prenyl bibenzyl derivatives) and bis-bibenzyls, the distribution of terpenoids is generally almost limited, with some exceptions for Portuguese species that appear to be a rich source of sesquiterpenoids [48]. On the other hand, 264 of 679 liverwort species so far examined proved to contain α-tocopherol, which antioxidative properties are assumed to be pivotal for the constituents of oil bodies of liverworts and supposedly the cause of its activity against plant pathogenic fungi [49].

Terpenes and terpenoids constitute a group of natural and biochemically active metabolites that have been found in all living organisms [50]. For example, steroids are essentials components of cell membranes in eukaryotic cells and carotenoids are pivotal in photosynthetic organisms. Therefore, it has been speculated that the terpene and terpenoid biosynthetic pathway might be very ancient. Bryophytes are also capable to synthesize a vast variety of terpenes and terpenoids and many of these metabolites resemble those observed in flowering plants, while others are peculiar of some bryophytes [42,51]. In particular, it has been reported that liverworts synthesize around 1600 terpenoids [5] and one reason of this high structure variety might lie in the high presence of oil bodies—discussed above—a distinctive cellular compartment of liverworts [52]. These intracellular organelles are surrounded by a single membrane layer and include proteins, lipophilic materials, some phenolic compounds (marchantins) and terpenoids [53]. Neither mosses nor hornworts possess these compartments, but they have the ability to produce different types of terpenoids as well. From a structural point of view, terpenes are simple hydrocarbons, while terpenoids (also called isoprenoids) are terpenes in which the structure has undergone rearrangements and modifications with the addiction of one or more oxygen residues. However, the terms “terpene” and “terpenoid” are often used interchangeably [54]. The classification of terpenoids is based on the number of carbon atoms included in the structure and in bryophytes the most common and characteristics are monoterpenoids (C10), sesquiterpenoids (C15), diterpenoids (C20) and triterpenoids (C30).

Monoterpenoids are peculiar metabolites that contribute to the characteristic fragrances of the plant material when crushed. In particular, sabinene, α-pinene, limonene and the acetate derivatives of borneol, nerol, geraniol and myrtenol are accumulated in liverwort [55], whereas the most mosses synthetize ß-citrocitral, α- and ß-pinene, limonene, camphene and bornyl acetate [42]. For some of them, an antifungal activity is well documented [56]. The few data reported for the hornworts suggest that limonene is the most abundant monoterpenoid produced in *Anthoceros caucasicus*, followed by α- and ß-pinene, myrcene and terpinolene [57].

**Figure 1:**
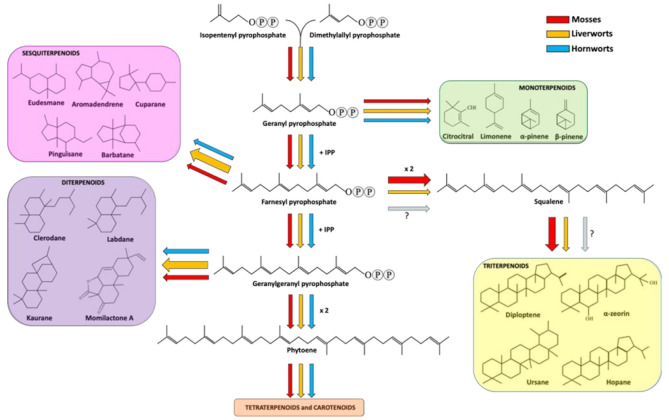
Terpenoid biosynthetic pathway in bryophytes. Red, orange and cyan arrows indicate the metabolic fluxes observed in mosses, liverworts and hornworts, as suggested in various reports (see references in the main text). Arrow thickness represents the amount of carbon streams (resources) committed for the production of specific metabolites in a specific bryophyte division. The question marks next to the faint arrows state that no information is available about the production of squalene and triterpenoid in hornworts.

Sesquiterpenoids includes three isoprene units and display a variety of forms, spacing from linear to mono-, bi- and tricyclic structures. Sesquiterpenoids represent the widest group of terpenoids produced by bryophytes. As well explained in a recent review [44], liverwort produces around 900 types of sesquiterpenoids deriving from 60 different skeletal groups. Eudesmane and aromadendrene structures are the two most representative backbones, followed by cuparane, pinguisane and barbatane [42] (Figure 1).

A peculiarity of liverwort sesquiterpenoids is that many are enantiomers of those found in seed plants [5], with few exceptions regarding germacrane- and guaiane-type sesquiterpenoids [43]. For example, it was seen that both *Conocephalum conicum* (liverwort) and *Leptospermum scoparium* (angiosperm) are able to produce cadina-3,5-diene, but the former synthesizes the (+)-enantiomer, whereas the latter the (-)-enantiomer [58]. Moreover, frullanolide and β-caryophyllene can be found in both enantiomeric forms in the liverworts *Frullania tamarisci* and *Pellia epiphylla*, respectively [59,60,61]. Another interesting feature of some liverwort sesquiterpenoids lies in the occurrence of these metabolites in nature: in fact, while some sesquiterpenoids such as the pinguisanes were only found in liverworts [42], others, despite appearing also in other organisms, are almost rare (such as 1,10-seco- and 2,3-seco-aromadendranes and africane-type sesquiterpenoids [6,42,55,62,63]). Finally, some sesquiterpenoids are liverwort species-specific [5]. In mosses, the number of sequiterpenoids was estimated around 100, a value substantially lower than that encountered in liverworts [42]. A possible explanation, beside the presence of oil bodies in liverworts, is that liverworts have been much more studied than mosses, resulting in a deeper knowledge of the chemical composition. Sesquiterpenoids are also produced in hornworts, as reported by Sonwa and Konig (2003) [57], but the available information on this division is still almost limited. Among the sesquiterpenoids, Neves and coworkers (1999) investigated the ability of *Targionia lorbeeriana* (a liverwort) extracts against two fungal pathogens: *Cladosporium cucumerinum* and *Candida albicans* [64] The former infects many species belonging to the Cucurbitaceae family, whereas the latter is a well-known animal pathogen. In that report, three guaianolide-type sesquiterpene lactones and two germacrane-type sesquiterpene lactones were extracted, isolated and tested, revealing that the main sesquiterpene lactone constituent of *T. lobeeriana*, the dehydrocostus lactone, was particularly effective against *C. cucumerinum*. Moreover, the acetyltrifloculoside lactone and 11-αH-dihydrodehydrocostus lactone exerted antifungal activity towards *C. cucumerinum* as well, even in a less extent. On the other hand, among the five tested compounds, only the 8,15-acetylsalonitenolide (a germacranolide) showed activity against *C. albicans*. The fifth compounds, i.e., the 8-acetylsalonitenolide (a germacranolide), did not show any biological activity in the bioassays. In the report of Scher et al. (2004), different compounds extracted from *Bazzania trilobata* (a liverwort) were tested against different fungal pathogens, such as *Botrytis cinerea*, *Cladosporium cucumerinum*, *Phythophthora infestans*, *Pyricularia oryzae* and *Septoria tritici* [65]. More in detail, six sesquiterpenes, such as 5- and 7-hydroxycalamenene, drimenol, drimenal, viridiflorol and gymnomitrol, showed different growth inhibitor activities. 5-hydroxycalamenenes was particularly effective in the inhibition of *P. oryzae*, whereas the 7-hydroxycalamenenes towards all the pathogens. Furthermore, this last was also tested in vivo in a glasshouse environment and it showed a significant reduction in the infection of grape vine leaves caused by *Plasmopara viticola* when used at 250 ppm. Drimenol was active against *C. cucumerinum*, whereas the aldehydic form, i.e., drimenal, showed a strong inhibiting activity against *S. tritici* and *P. infestans*. Viridiflorol showed weak activity against *C. cucumerinum* and *P. oryzae*. Gymnomitrol exerted strong activity against *P. infestans* and moderate against *C. cucumerinum*, *P. oryzae* and *S. tritici*.

Diterpenoids are specialized metabolites deriving from the condensation of four isoprenyl units. They are the second largest group of terpenoids accumulated in liverworts. More in detail, about 500 compounds have been reported in liverworts so far, including acyclic, di-, tri-, tetra- and pentacyclic diterpenes. From a structural point of view, the most part of diterpenoids are based on clerodane, kaurane and labdane skeletons [5,42] (Figure 1). Interestingly, succulatane, infuscane, *abeo*-labdane, spiroclerodane, 5,10-*seco*-clerodane or 9,10-*seco*-clerodane backbones were found to be liverwort-specific [42]. In mosses, the number of identified diterpenoids is lower and non-exclusive of this division. For example, the momilactone diterpenoids extracted from *Hypnum plumaeforme* (moss) were also found in rice [66]. As for sesquiterpenoids, diterpenoids were also found in hornworts [57]. Among the diterpenoids, momilactones A, B and F, and acrenol were extracted from the moss *Hypnum plumaeforme* Wilson and they strongly inhibited the growth of the fungal pathogens *P. oryzae*, *Colletotrichum acutatum* and *Ustilago maydis* [67]. More in detail, the growth of the rice pathogen *P. oryzae* was inhibited by momilactones A and B, with the latter showing a greater efficacy against spore germination and germ tube growth of the fungus [68]. Momilactone A and B activities were also investigated on other fungal pathogens, giving encouraging results in the inhibition of *B. cinerea*, *Fusarium solani* and *Colletrotrichum gloeosporioides* [69].

Triterpenoids derive from the condensation of six isoprene units. In liverworts, the most common triterpenoids are hopanoids, including diploptene, diplopterol and α-zeorin [5,42]. In comparison to mono-, sesqui- and diterpenoids, mosses are richer in triterpenes, including ursane, fernane, friedelane, hopane, lupane, taraxane, cycloartane, obtusifolane, dammarane, polypodane and serratane [5,42]. No data are available about triterpenoids in hornworts so far.

### 3.2. Phenolic Compounds 

Bryophytes are considered a phylogenetic divergent group from the most evolved vascular plants. These are organisms able to synthesize lignin, a polymer that results from the polymerization of monolignols and it is one of the final products of the phenylpropanoid pathway. Lignin and lignans, which share monolignols as common precursors, are both involved in the plant defense against pathogens, often resulting fungal elicitors [70]. In bryophytes, the presence of candidate orthologous genes encoding for enzymes involved in the production of the monolignols p-coumaryl alcohol and coniferyl alcohol (that has been reported to act as a fungicide against *Ceratocystic* spp. pathogens [71] and is effective in inhibiting the growth of *Verticillium longisporum*) in *Physcomitrella patens* has been reported [72,73,74] but true lignin has never been detected, meaning that at least part of the biosynthetic machinery involved in lignin formation is present in these plants [75,76,77].

**Figure 2:**
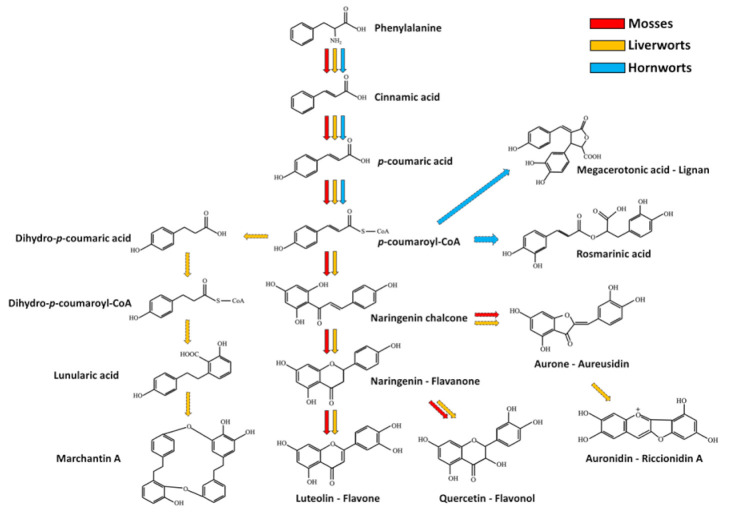
Simplified phenylpropanoid pathway and some of its putative branches in bryophytes. Red, orange and cyan arrows indicate the metabolic fluxes observed in mosses, liverworts and hornworts respectively, as suggested in various reports (see references in the main text). Dashed arrows indicate multiple reaction steps between the precursor and product.

Monolignols are also precursors of lignans that were found in several bryophytes and some of them are currently used as chemical markers in chemosystematics. For example, megacerotonic and anthocerotonic acids are peculiar lignans of hornworts [42], and show a relationship to the antifungal defense compound rosmarinic acid [78]. A simplified scheme representing the phenylpropanoid pathway and part of its branches in bryophytes is depicted in Figure 2.

The phenylpropanoid pathway is the initial way for the production of other metabolites in flowering plants, such as benzoic acid, phenylpropanoid esters, hydroxycinnamic acids (including differently esterified and/or etherified acids), acylated polyamines, phenylpropenes, flavan-3-ols, aurones, stilbenes, isoflavonoids, flavonoids and coumarins [79]. In bryophytes, the production of different phenolic compounds belonging to some of the previously mentioned classes has been reported [80,81]. For example, hydroxycinnamic acid derivatives, such as methyl p-coumaric acid and caffeic acid methyl ester, have been detected in hornworts [82] and rosmarinic acid (Figure 2) and its glucoside [83]. Again, 4-O-caffeoylquinic, 5-O-caffeoylquinic and caffeic acids were detected in the two mosses *Brachytheciastrum velutinum* and *Kindbergia praelonga* [77]. Besides these compounds, bryophytes are known to produce derivatives of benzoic acids [42,84], stilbenes [85], coumarins [86,87] and different flavonoids, including flavones (apigenin and luteolin derivatives for example) [77]; flavonols (quercetin) [77], isoflavones [88], dihydroflavonols [89] and aurones [90,91]. Interestingly, genes involved in phenylpropanoid-polyamine biosynthesis are not found in bryophytes [92]. Phenylpropanoids have been found to be important for the response of *Arabidopsis thaliana* against some fungal pathogens [93]; at the same time, the phenylpropanoid biosynthetic pathway has been shown to be activated after the assault of different pathogens in mosses. For example, the two fungal pathogens *B. cinerea* and *Colletotrichum gloeosporioides* triggers the activation of genes involved in phenylpropanoid production in *P. patens* [94,95,96], thus suggesting a role of these substances in plant protection. Moreover, five aromatic esters isolated from *Balantiopsis cancellata*, including 2-phenylethyl (Z)-cinnamate and 2-phenylethyl (E)-cinnamate, showed antifungal activity towards *Cladospirum herbarum* [97]. Finally, isoprenyl benzoates found in the liverwort *Trichocolea tomentella*, such as tomentellin and demethoxytomentellin, and trichocolein, isolated from *Trichocolea lanata*, displayed antifungal activity against *C. albicans* and *Trichophyton mentagrophytes* [12].

**Figure 3:**
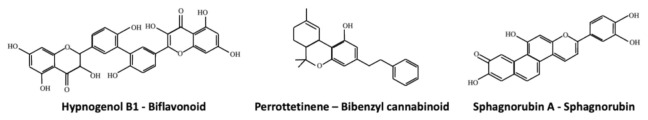
Examples of the biflavonoid Hypnogenol B1, the bibenzyl cannabinoid Perrottetinene and the Sphagnorubin A.

In comparison with flowering plants, many phenolic compounds are found in a multimeric form in bryophytes. Bibenzyls, bisbibenzyls and bisbibenzyl dimers are peculiars of liverworts [98]. Marchantins, riccardins, plagiochins, bazzanins and lunularic acid are only some examples of this molecule class [42,99]. Marchantin A (Figure 2) was the first characterized member of cyclic bis(bibenzyls) molecules found in the liverwort *Marchantia polymorpha* [6,100] and its synthesis was proposed to occur through the condensation of monomeric bibenzyl precursors, such as lunularic acid (Figure 2) and lunularine [60]. Moreover, other compounds seem to be involved in marchantin A production, such as L-phenylalanine, acetate, malonate, cinnamic acid, p-coumaric acid and dihydro-*p*-coumaric acid [101]. Marchantin A from *M. polimorfa* and marchantin C, neomarchantins A and B from *Schistochila glaucescens* were reported to be active against the dermatophytic fungus *Trichophyton mentagrophytes* [102,103]. Riccardin A, isolated from *Riccardia multifida* [104,105], and plagiochin A (a macrocyclic bisbenzyls, Figure 3), characterized from *Plagiochila sciophila* [106], are other macrocyclic bisbibenzyls, and some related compounds, such as riccardin D, riccardin B, asterellin A, asterellin B, 11-demethylmarchantin I, dihydroptychantol, marchantin H, marchantin M and marchantin P showed antifungal activity against *C. albicans* [107,108]. Plagiochin E from *M. polimorfa* was also found to inhibit the growth of *C. albicans* as well, by negatively affecting the cell wall chitin synthesis [109]. The linear bisbibenzyl perrottetins E was detected in *Radula perrottetii* [105], while the bisbibenzyl dimer pusilatin A was isolated from *Blasia pusilla* [110]. Lunularic acid was isolated from *Lunularia cruciata*: it promotes dormancy and inhibits growth in liverworts, and, interestingly, it displays a weak antifungal activity [111]. Among the bibenzyls, it is worth to recall that *Radula* species accumulate rare bibenzyl cannabinoids [42,63] (Figure 3). Regarding the antifungal activity, the three polychlorinated bibenzyls 2,6-dichloro-3-hydroxy-4′-methoxybibenzyl, 2,6,3′-trichloro-3-hydroxy-4′-methoxybibenzyl and 2,4,6,3′-tetrachloro -3,4-dimethoxybibenzyl extracted from some *Riccardia* sp. have been shown to exert an effect against the growth of *Cladosprium herbarum* [97]. Moreover, three bisbibenzyls, such as bazzanin B, isoplagiochin D and bazzanin S, isolated from *Bazzania trilobata* have been shown to moderate inhibit the growth of *B. cinerea*, *Cladosporium cucumerinum*, *P. infestans* and *S. tritici*, whereas the strongest activity was observed for *M. oryzae* [65]. Finally, marchantin A isolated from *Marchantia* sp. showed antifungal activities against several fungi, including *Alternaria kikuchiana* and *Rhyzoctonia solani* [9].

Biflavonoids and triflavonoids are typical of mosses [42,99,112], whereas liverworts mainly produce monoflavonoids, which are usually C-glycosides and O-glycosides. For example, the two mosses *Hypnum cupressiforme* and *Bartramia stricta* produce the biflavonoids hypnogenol B1 (Figure 3) and 5′,3‴-dihydroxyamentoflavone, respectively, whereas the liverwort *Lunularia cruciata* is able to synthesize luteolin-7-O-glucoside and quercetin [77]. Finally, the synthesis of the triflavonoid aulacomniumtriluteolin has been reported in *Aulacomium paustre*. It has been suggested that biflavonoids might negatively affect the growth of fungal pathogens since extracts of the moss *Hypnum cupressiforme* showed antifungal activity and these extracts were rich of polycyclic aromatic hydrocarbons, hypnogenols, biflavonoids and hydroxyflavonoids [113,114,115].

The ability to generate multimeric forms of flavonoids is considered as a higher evolved character in mosses. In particular, it has been reported that the moss *Takakia lepidozioides*, which is considered one of the most primitive moss, synthesizes only monoflavonoids, including flavone O-glucuronides, flavone C-glycosides and flavonol O-glycosides [116], whereas more evolved mosses mainly produce bi- and tri-flavonoids. Interestingly, hornworts do not synthesize flavonoids [117,118,119]. It was hence suggested the hypothesis that the flavonoid biosynthetic pathway might be absent in hornworts due to an earlier phylogenetic divergence from other land plants, in which this feature developed later. However, it cannot be excluded that flavonoid genes might have been lost during the evolution in hornwort. Further studies are needed to clarify this aspect. Bryophytes also produce pigmented substances: aurones have been isolated from both mosses and liverworts [91,120], auronidins (Figure 2) from *Marchantia* [120] and sphagnorubins (Figure 2) from *Sphagnum* species [121]. Auronidins were initially associated to anthocyanidins, due to the similar pigmentation provided; for example Riccionidin A (Figure 2), an auronidin associated to the cell wall of the liverwort *Ricciocarpos natans* [122], was found to be responsible for its red pigmentation. However, a substantial difference in the chemical nature of auronidins and anthocyanidins has been recently reported: in fact, although the initial steps of the flavonoid pathway are in common, the final part of the biosynthetic pathway might be different; additionally, auronidins possess fluorescence properties stronger than anthocyanidins [120]. Unfortunately, at the best of our knowledge, no information is available in literature regarding the antifungal property of these pigmented compounds.

### 3.3. Other Compounds

**Figure 4:**
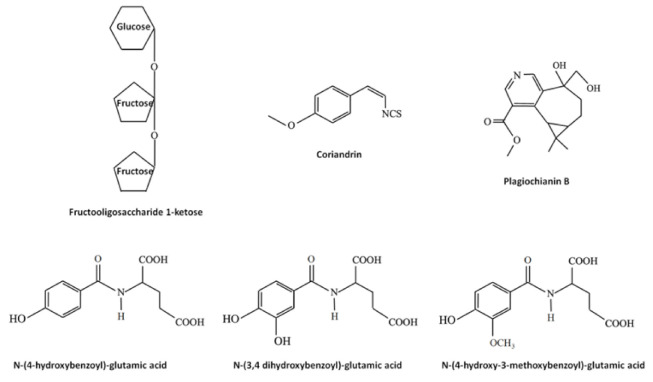
Some example of other molecules produced in bryophytes and three having antifungal effects, such as the *N*-(4-hydroxybenzoyl)-glutamic acid, *N*-(3,4 dihydroxybenzoyl)-glutamic acid and *N*-(4-hydroxy-3-methoxy-benzoyl)-glutamic acid.

Bryophytes produce and accumulate a vast array of other molecules exerting biological effects, including saccharides, lipids, vitamins and nitrogen- and sulfur-containing compounds. Some examples are reported in Figure 4. Beside the traditional saccharides, for example sucrose, which can be found in both flowering plants and bryophytes, tri-, oligo- and polysaccharides, such as the rare fructooligosaccharide 1-ketose extracted from the moss *Rhodobryum ontariense* and the liverwort *Porella platyphylla* [123,124], are unique for bryophytes. Lipids found in bryophytes include (i) membrane lipids, such as sterols (in particular, in higher evolved mosses have been found stigmasterol, campesterol and sitosterol), fatty acids and glycerol-, phosphor- and sphingo-lipids; (ii) storage lipids, such as triacylglycerols and sterol esters; (iii) surface lipids involved in the formation of part of the cuticle, such as cutin and waxes and (iv) signaling lipids, including phosphoinositides and oxylipins [125]. Interestingly, mosses are known to produce long unsaturated fatty acids, such as the eicosanentaenoic and arachidonic acids [24]. Vitamin E (α-tocopherol) and K were found in liverworts and it was demonstrated that, among 700 liverworts, almost all contained α-tocopherol and squalene [126]. It should be noted that, in seeds oil, tocopherols have been indicated as the major antifungal principles, due to their antioxidant properties [49]. Nitrogen- and sulfur-containing compounds are almost rare in bryophytes. In liverworts, for example, the nitrogen- and sulfur-containing compounds coriandrins (isothiocyanates, Figure 4) are present in *Corsinia coriandrina* [127], and the nitrogen-containing alkaloid plagiochianin B (Figure 4) from *Plagiochila duthiana* [128] and two prenylated indole derivatives from *Riccardia* species [129]. In mosses, another nitrogen-containing alkaloid (harmol), active against both the phytopathogenic fungi *Penicillium digitatum* and *Botrytis cinerea* [130], was reported from *Fontinalis squamosa* [131]. In hornworts, *Anthoceros agrestis* was found to contain nitrogen-containing molecules such as glutamic acid amide derivatives of hydroxybenzoic-, protocatechuic-, vanillic, isoferulic and coumarylic acids [132], some of them with a well characterized inhibitory activity against fungal pathogens [133,134,135]. *N*-(4-hydroxybenzoyl)-glutamic acid, *N*-(3,4 dihydroxybenzoyl)-glutamic acid and *N*-(4-hydroxy-3-methoxy-benzoyl)-glutamic acid structures are showed in Figure 4.

## 4. Mining with the Omic-Technologies: New Generation Approaches That Contributed—and Still Do—to the Individuation of Antifungal Bryo-Actives

The exploration of new molecular targets for antifungal compounds has generated considerable research, especially using modern omics methods (genomics, genome-wide collections of mutants and proteomics) and bioinformatic approaches. Recently, micro- and nanoscale strategies have been introduced in the drug discovery focused on antifungals. Microfluidic platforms have been developed, since they possess several advantages if compared to traditional multiwell-plate screening: among them, the low reagents consumption, the possibility to simultaneously and independently manipulate a huge number of samples, and the ease of integrating various analytical standard operations and large-scale integration, are the most attractive [136]. Despite a low morphological complexity, bryophytes generally display a high degree of chemical diversification [42,63,137], which reflects specific diversification at the genetic level. In this section, we show how, at a molecular level, the in depth analysis offered by the meta-omics approaches (which mainly include metagenomic, transcriptomic, proteomic and metabolomic studies) resulted in being highly informative in the case of bryophytes and it is already acknowledged for higher plants, with particular regard to the functional diversity of antifungal bioactive compounds.

### 4.1. Genomics and Transcriptomics

The functional genomics approaches, aided by targeted-metabolite and transcriptome profiling, represented a highly effective way to deciphering novel gene functions involved in specialized metabolic pathways in medicinal plants, enabling not only drug discovery, but also drug development and large-scale production of phytomedicinals. Although several bioactives (such as antifungal and antimicrobial compounds, including enzymes) are reported from bryophytes, the characterization of most of them at a genetic level has not been achieved yet; in fact, despite the relatively low cost of sequencing, de novo assembly of whole genomes without prior sequence information is still costly, and requires intensive computational resources [138].

Even after 15 years of next generation sequencing (NGS) technology availability, genome sequence of only seven bryophyte have been reported to date: (i) the nuclear genome of the desiccation-tolerant moss *Physcomitrella patens* (480 Mbp; 35,938 protein-coding genes), which was firstly sequenced in 2008 and then reannotated in 2013. The data provide an excellent resource for functional and comparative genomics, since a large number of knockout lines are available at the International Moss Stock Center (http://www.mossstock-center.org/) [139,140]; (ii) the genome of the liverwort *Marchantia polymorpha* (225.8 Mb; 19,138 protein-coding genes), released in 2017 [141]; (iii) the genome of the dioicous moss *Ceratodon purpureus* (362.5 Mb; 19,138 protein-coding genes; *Ceratodon purpureus* GG1 v1.1 DOE-JGI; http://phytozome.jgi.doe.gov/) [142] and (iv) the genome of the hornwort *Anthoceros angustus* (148 Mb, 14,629 protein-coding genes) [143]. More recently, annotated genomes of (v) *M. paleacea* (238.61 Mb), (vi) *M. polymorpha* ssp. *polymorpha* (222.7 Mb) and (vii) *M. polymorpha* ssp. *montivagans* (225.7 Mb) have been also reported Radhakrishnan et al. (2020) [144]. Currently, only four complete chloroplast genomes are available, sequenced in *Physcomitrella patens* (122,890 bp) [145], *Tortula ruralis* (122,530 bp) [146], *Tetraphis pellucida* (127,489 bp) [147] and *Tetraplodon fuegianus* (124,374 bp) [148].

Acquiring knowledge on the bryophyte genomes is critical not only to clarify their evolutionary and phylogenetic relationship with vascular plants, but also to identify genes potentially involved in the biosynthesis of antifungal (when not fungal-interaction modulating) substances. For example the two genomic regions Fungal Region 1 (FR1) and Fungal Region 2 (FR2), identified in the nuclear genome of *Physcomitrium patens*, proved to contain mostly fungi-specific genes and mobile genetic elements [149]: based on sequence similarity, both regions were found to contain genes that could be roughly classified into three families encoding, respectively, a heterokaryon incompatibility protein (HET) domain that reportedly functions in the self/non-self recognition system of filamentous fungi), a HopQ1-like protein (HLP) that may act as a virulence factor in bacteria (Pp3c13_910), and a conserved hypothetical protein with unknown function but identified as a conserved fungal gene (CF). Strikingly, all these gene families (CF, HLP and HET domain-containing genes) appear to be functionally related to fungal interactions with other organisms, either different fungi or host plants. The authors speculated that, albeit some genes could been coopted by mosses for activities not directly related to interactions with fungi, some other fungi-derived genes might have been recruited to regulate, or counteract, the activities of same fungi against the plant, in addition to their roles in other processes.

To the so-called “resurrection plants” (a group of land plants tolerating extreme dehydration of their vegetative tissues and still quickly regain normal physiological and metabolic functions after rehydration) commonly are ascribed various bryophytes species, while rarely Pteridophytes and Angiosperms are found and no gymnosperms are represented [150]. Adaptation to extreme dehydration supposedly relies on the development of unique molecular mechanisms to protect these plants against desiccation-induced damage, including the expression of stress-protective genes and high abundance of protective metabolites [151]. Nevertheless, sequencing the genomes of bryophytes belonging to the could not be always achieved without complications, due to the large size, the lack of detailed genetic maps and the presence of repetitive sequences, that make straightforward annotations challenging [152]. Although the primary interest in resurrection species has been fuelled by their ability to withstand desiccation and, in turn, to their potential as a source for targeted gene discovery [153,154,155,156], the unique metabolites that frequently characterize such species have recently attracted much more attention with respect to potential uses in biotechnology and medicine; for example, Amentoflavone isolated from *Selaginella tamariscina* has strong anticancer/proapoptotic, antibacterial and antifungal activities [157,158,159]. If compared with whole genome sequencing, de novo transcriptome analysis has made it possible to unravel the genetic architecture of those organisms for which a reference genome was not available yet, at a low cost and computationally less intensive. The analysis of randomly selected cDNA clones or expressed sequence tags (ESTs), given this name because they represent only genes expressed at a particular time or under a particular circumstance, has been an important technique for the discovery of new genes [160]. Nevertheless, very few transcriptome data addressed to identification of antifungal compounds have been reported in bryophyte, while most of the works were aimed at identifying abiotic responses. This powerful technique was applied to investigate, at the transcriptomic level, the response of *P. patens* treated with abscisic acid [161] or cytokinin [162], and the response of *T. ruralis* to desiccation [163]. The published moss EST databases are relatively small, as 253 ESTs derived from *P. patens* and 152 ESTs from *T. ruralis* with the majority of the ESTs (52% and 71%, respectively) showed no significant similarity to previously characterized genes. More recently, “The 1000 plants”, an international multidisciplinary consortium generating large-scale gene sequencing data for over 1000 species of plants, reported 55 bryophyte transcriptomes, from 41 sample of mosses and 14 samples of hornworts (oneKP, or 1KP; https://db.cngb.org/onekp/) [164,165].

Among secondary metabolites involved in abiotic stress, terpenoids have long time been recognized to play an important role in bryophyte, and in vascular plants, environmental interactions [5,136]: in fact, over the last four decades, more than 1600 compounds belonging to the terpenoids family have been reported from this plant lineage [6,42,60], a number of them demonstrating the ability to possess antioxidant, antimicrobial and cytotoxic activities that suggest a potential for pharmaceutical purposes—mainly addressed to emerging drug resistance occurrences. In this scenario, Singh et al. [166] analyzed, in 2015, the transcriptome of the thalloid liverwort *Dumortiera hirsute*, highlighting a total of 33, 662 unigenes; afterwards searched for various pathways involved in the *D. hirsuta* metabolism, the transcriptome revealed 95 significant pathways. In particular, amongst the 721 unigenes constituting metabolic pathways, 309 unigenes were relevant to secondary metabolites biosynthesis. A strong representation of genes for terpenoid (29 genes), abscisic acid (28 genes) and flavonoids biosynthesis (10 genes) were observed. These were supposed to be the major components of both biotic and abiotic stress tolerance, and thought to be involved in cell signaling [167].

A transcriptomic approach was also used to screen RNA samples from *M. polymorpha* thalli, by using Illumina Hiseq^TM^ 2000 instrument. Marchantin A, the first characterized macrocyclic bis(bibenzyls) found in this liverwort, showed interesting antifungal activities [42,60,137]. Moreover, Friederich et al., (1999) [101] reported the phenylpropane/polymalonate pathway in the biosynthesis of the marchantins in *M*. *polymorpha.* Thus, to clear this pathway, transcriptome sequencing and digital gene expression analyses of *M*. *polymorpha* were carried out by using the Illumina RNA-seq system [168]. Transcriptome de novo assembly for the identification of novel genes responsible for the biosynthesis of secondary metabolites was also performed on *Radula marginata* in the absence of a reference genome [138]. Due to this investigation, a structural analog of tetrahydrocannabinol (19-THC)—a psychopharmacological compound belonging to the class of terpenophenolics typical of *Cannabis sativa* L.—was found, and, even if cannabinoids have been widely characterized in different plant species, this was the first report of similar compounds in an early plant. Cannabinoids have a wide-ranging role in numerous clinical applications, which exploit their antimicrobial and antifungal capabilities (in addition to a biological effect on insects and mollusks, piscicidal properties, antileishmanial and antitrypanosomal and cytotoxicity and anti-inflammatory activities [169]. Thus, the individuation of such a metabolite in *R. marginata* results in being of great importance, since, because of the relatively simple architecture and the diversity of its natural habitats, this plant could be suggested as an alternative source of earning. 

Even though research about the regulation of the plant–pathogen relationship in early-diverging land plants has become more popular in different areas, the underlying mechanisms and interactions between many transcription factors and their associated stress-responsive genes are not fully understood yet, especially at the ancestral state. Recently, Carella et al., (2019) [41] defined the transcriptional and proteomic response of the early-divergent *M. polymorpha* to infection with the pathogenic oomycete *Phytophthora palmivora.* To gain further insight into the classes of *M. polymorpha* loci responding to *P. palmivora* infection, the analysis was focused on RNA-seq and proteomics datasets on the annotations, and phylogenetic analyses of conserved land plant gene families, described by Bowman et al. (2017) [141]. In this case, authors revealed different responding sets of *Marchantia* gene families, including those associated with transcriptional regulation, the cell wall and cuticle, hormone biology, phenylpropanoid (flavonoid) biosynthesis, lipid peroxidation, terpene synthesis, vesicular trafficking, transporters and membrane H^+^-ATPases, kinases and receptors already described in these plants [97,170]. Taken together, the results demonstrated that the *Marchantia* response to oomycete infection displayed evolutionarily conserved features indicative of an ancestral deterrence strategy against fungal pathogens and based on phenylpropanoid-mediated biochemical defenses. A comparative RNA-seq analysis conducted on the angiosperm *Nicotiana benthamiana* interested by *Phytophthora* infection, let to the discovery of groups of pathogen-responsive genes orthologous in *Marchantia* and *Nicotiana*, which included genes encoding for enzymes associated with phenylpropanoid and flavonoid biosynthesis such as phenylalanine ammonia lyase (PAL), chalcone synthase (CHSs), chalcone isomerase (CHI-like) and cinnamate 4-hydroxylase (C4Hs), resulted in being upregulated in this condition. More importantly, the same genes found in oomycete-colonized *N. benthamiana* were shown to be similarly induced in moss treated with microbial elicitors [171,172] or infected with *Pythium irregular* [173]. Additionally, these data also suggested a role for a phylogenetically basal R2R3-MYB transcription factor, MpMyb14, in mediating the flavonoid-associated biochemical defenses during pathogen infection in *M. polymorpha* thalli. In particular Mpmyb14 mutants, which are defective in activating phenylpropanoid biosynthesis gene expression, exhibited an enhanced susceptibility to *P. palmivora* infection, whereas the ectopic overaccumulation of MpMyb14-regulated pigments dramatically suppressed pathogen growth in planta, suggesting a protective role for these compounds during biotic stresses. 

### 4.2. Proteomics

In recent years, advances in the field of DNA sequencing of plant species have made it possible to undertake proteomics studies, as this allowed the creation of increasingly complete protein databases. Although there are still many limitations due, for example, to the occurrence of post-translational modifications and the availability of genomic data for a limited number of species, the combination of proteomics with the other omics sciences is pivotal to a deeper understanding of biological processes, being valid for vascular plants and early plants. Various studies conducted on moss *P. patens* have highlighted the importance of this species to study mechanisms such as biotic and abiotic stress, or adaptation, critical for the environmental interaction of plants, and to characterize stress-related genes also through reverse genetics approaches [174]. For example, due to the possibility to cultivate *P. patens*, under standardized conditions, in bioreactors or bubble flasks [175], an adequate amount of plant material was achieved, allowing to perform a proteomic investigation that resulted in being strategic to highlight the importance of protein secretome as a key player in cell signaling and cell-to-cell communication in responses to fungal pathogens elicitors [176]; this study demonstrated how the secretome of the moss was dominated by pathogen defense-related proteins when plants were grown in liquid culture and treated with chitosan: a strong oxidative stress was found as correlated with secreted peroxidase Prx34, a peroxidase involved in defense against moss pathogens [177,178]. Authors exploited a shotgun approach, chromatography associated with tandem mass spectrometry (LC–MS/MS), using an Orbitrap spectrometer coupled with Proxeon EASY-nLC system, for the identification of extracellular proteins, while precipitation was analyzed by two-dimensional electrophoresis (2-DE). Of the secreted proteins identified 70% were found to be homologous to those secreted and characterized in Tracheophytes, some of them belonging to the functional categories correlated to defense against pathogens; interestingly, moss treated with chitosan presented 72 unique proteins that were not found in the control samples: among them, chitinases and thaumatin were recognized, as frequently observed in higher plants exposed to pathogens or defense-related elicitors. It should be noted that industrial applications have been reported for proteins present in the filtrates of these cultures, in the production of recombinant protein-based antifungal formulations [175].

Similar shotgun proteomic method was also applied in the above mentioned study investigating the response of liverwort *M. polymorpha* to *P. palmivora* [41], using an EASY-nLC 1200 coupled to an Exactive Plus Q mass spectrometer and a MaxQuant software (version 1.5.7.4, http://www.maxquant.org/) [179] with unlabeled quantification (LFQ) and iBAQ enabled for mass spectra processing. Protein identification was conducted using a combined database of *M. polymorpha* transcriptome sequences (http://marchantia.info) [141].

### 4.3. Metabolomics

In the last 10 years, several studies have been carried out on natural extracts from different bryophytes, aimed at the characterization of interesting metabolites: arrays of low molecular weight molecules constituting the “metabolic phenotype” (metabolome) of selected organisms have been screened defined. The separation of most of these compounds can be conducted using reverse phase liquid chromatography on a C18 column, coupled to a high resolution mass spectrometer (QTOF-MS) through an atmospheric pressure ion source (ESI or APCI). This technology proved to be effective in obtaining information on the structure through the fragmentation of the analyzed compounds, due to the presence of a quadrupole segment and a collision cell before the flight tube. The bioinformatic analysis of metabolomic data (both LC/MS and GC/MS) requires the aid of tools that allow the extraction of chromatographic and spectral data in a file format such as xcms [180], metAlign [181], MzMine [182] or GridMass [183]. Tools for integrated GC/MS analysis are also available, that allow one to perform peak alignment, RI calculation and MS spectra matching in databases such as TargetSearch, TagFinder or FiehnLib. Derivatization is reported to be not necessary for the analysis of volatiles and, due to the nature of the matrix, the detection limit can be very low [184].

As for the other non-model organisms, the greatest challenge is the identification of compounds due to the lack of constitutive reference spectra, the large number of completely unknown compounds. In fact, in the near past, only a few studies in the literature are found that performed untargeted metabolomics analyses (LC/MS, GC/MS and NMR) with bryophytes [185,186,187,188,189]. More recently, an integrative ecometabolomics approach, which proposed to connect biochemistry with ecology using bioinformatic methods, applied untargeted liquid chromatography coupled with ultra-performance liquid chromatography coupled to electrospray ionization quadrupole time-of-flight mass spectrometry (UPLC/ESI-QTOF-MS) to obtain metabolite profiles of methanolic extracts from nine bryophyte species (namely *Brachythecium rutabulum*, *Calliergonella cuspidata*, *Fissidens taxifolius*, *Grimmia pulvinata*, *Hypnum cupressiforme*, *Marchantia polymorpha* L., *Plagiomnium undulatum* (Hedw.) T.J. Kop., *Polytrichum strictum* and *Rhytidiadelphus squarrosus*), in order to unravel their metabolic variation across seasons, as both their growth and biochemistry are strongly dependent on the season: as a result, typical secondary metabolites possessing antimicrobial and antifungal properties, such as apigenins, lunularic acid, lunularin, various marchantins and perrottetin E, were identified, but other compounds belonging to the classes of methoxyphenols, neolignans, cinnamic acids, lactones and anthocyanins, active against fungal pathogens, were annotated [45,190]. Metabolite profiling of *Sphagnum fallax* performed using two types of mass spectrometry (MS) systems and ^1^H nuclear magnetic resonance spectroscopy (^1^H NMR) revealed a total of 655 metabolites—329 of which were novel metabolites. Authors compared *Sphagnum’s* metabolite signature to the metabolite composition of different plant species with known antimicrobial effects (i.e., medicinal plants), finding 17 putative antimicrobial substances: network analysis identified 211 known compounds and 21 unknown compounds that were first neighbors of these 17 antimicrobial compounds, suggesting there are potentially other compounds in the *Sphagnum* metabolome with antimicrobial properties. Other compounds known for their potential against fungi as phenolic compounds, benzoic, caffeic and coumaric acids derivatives, have been detected in a study carried out by HPLC-TOF/MS on *Cryphaea heteromalla* extracts [84].

Among all metabolites in bryophytes, lipids are considered one of the most important groups, as they play many vital roles in energy storage, membrane formation, cell signaling, functioning and environmental adaption [191]; they include all non-hydrophilic compounds but overall terpenoids, that due to their antimicrobial and anti-inflammatory potential are considered the reason of the ethnobotanical used in traditional medicine [24,192,193]. Lipidomics, the emerging field for the high-throughput analysis of lipid-related metabolic pathways in animals and in plants, essentially relies on the screening of biomarkers by untargeted lipid analysis using liquid chromatography–mass spectrometry (LC–MS), which allows for the separation and identification of possible lipophilic compounds and derivatives. LC-based lipidomics is relatively rapid and usually requires less sample than GC-derived methods, since only 2–10 mg of leaf dry weight is needed, but these advanced techniques only recently have been applied on bryophytes extracts: in 2017, Gacet and coauthors employed targeted metabolomics using LC–MS/MS and GC–MS to study signaling lipids in 71 plant species, representative of major phylogenetic clades, to systematically investigate the distribution of lipids in the plant kingdom; they found a fascinating positive phylogenetic correlation in the jasmonic acid (JA) abundance of early plants versus Angiosperms and Gymnosperms [194]. JA and its derivatives, jasmonates, belong to the class of oxylipins, bioactive metabolites derived from the oxygenation of acidic polyunsaturated fats [195]: besides primarily exerting regulatory functions involved in plant reproductive development and vegetative growth, jasmonates not only play critical roles in the defense response against wounds and attacks by pathogens, but also have been recently reported to possess a direct antifungal activity against a number of fungal phytopathogens [196]. Gachet et al. demonstrated that, while completely absent in algae, JA started to be detected in small to intermediate amounts in bryophytes (namely: hornworts *Anthoceros agrestis, Anthoceros punctatus* and *Phaeoceros laevis*; mosses *Physcomitrella patens, Funaria hygrometrica, Polytrichum juniperinum, Hedwigia ciliata* and *Hylocomium splendens* and liverworts *Conocephalum conicum, Marchantia polymorpha* and *Riccia fluitans*), with the ending to be more represented in higher plants. They therefore speculated that the phylogenetic distribution of JA, and other lipids, could be a consequence of interactions/adaptations of plants to the surrounding environment, including chemical interactions and defense mechanisms, not completely elucidated yet.

## 5. Most Common Techniques for the Validation of Antifungal Activity of Bryophyte Extracts/Compounds

From the 50s, a systematic literature about laboratory tests aimed to evaluate the biological properties of bryophyte extracts appeared, and, during the following five decades, aqueous and alcoholic extracts and isolated compounds from about 150 species of liverworts and mosses had been successfully tested against fungi and bacteria in in vitro assays [9,100,197,198,199,200,201,202,203,204,205,206,207]. In a complete report of several bryophytes extracts/purified compounds showing activity (or investigated) against human and plant pathogenic fungi published in 2011, the different modes of action of such bryoactives were described: among them, spore germination inhibition, development of anomalies in the hyphae, formation of flaccid cell wall and granulated cytoplasm, inhibition of chitin production and the induction of reactive oxygen species (ROS) associated with mitochondrial dysfunction were noticed [208]. The mechanism of activity has been reported as not only dependent on the compound, but also pathogen specific; in addition, same compound was reported to follow a different mode of action while inhibiting the same or different fungi.

The majority of such investigations were essentially based on agar plates tests, but it is a fact that when we review a more recent literature about the antifungal activity of compounds or plant extracts, we face a plethora of studies difficult to compare, because of the use of different non-standardized approaches, growth media, inoculum preparation techniques and size, treatment conditions and time of determination; if the most known and simple methods are the disk-diffusion and broth or agar dilution assays, basically aimed at testing the antifungal properties of plant-derived agents, other procedures such as the time-kill test and flow cytofluorometry have been described and applied to obtain more punctual information on the nature of the inhibitory effect (whether fungicidal or fungistatic, its time- or concentration-dependence, etc.) and the specific cell damage inflicted to the target microorganism. Owing to the fascination towards the properties of bryophyte bioactives, and with an eye on the multidrug-resistance occurrence in both agricultural and human relevant fungal species, it is important to develop a better understanding of the existing methods available for characterizing and/or quantifying the biological effect of extract or pure compounds for its applications in various fields. Therefore, in this review, we provided a panel of the in vitro and in vivo techniques suitable for evaluating the activity of natural compounds against fungi, along with some examples of employment.

### 5.1. In Vitro Methods

Spore germination assay: For the evaluation of bryophyte extracts on fungal spore germination the slide technique has been reported [209,210]. Bryophyte extract of the desired concentration is added as a film to the surface of dried slides or in the cavity of the slide using the hanging drop method [211]. The suspension of fungal spores is taken and spread over the film. In the controlled treatment, distilled water is added in place of spore suspension. Slides are then incubated at 25 ± 2 °C for 24 h. After incubation, slides are fixed in lactophenol cotton blue and observed microscopically for spore germination. Percentage spore germination is calculated recording the number of germinated spores/the total number of spores [212]. Hyphal length can also be measured with this assay after 8 h from inoculation [213].

Poisoned food technique: in this test, especially used for antifungal testing, potato dextrose agar (PDA) medium is mixed with the desired concentration of sterilized bryophyte extract and the mixture is poured into the sterilized Petri dishes [214]. The Petri dishes supplemented with the same amount of distilled water instead of extract represent the control. After the complete solidification of the medium, mycelium plugs from five-to-seven days-old fungal culture were inoculated aseptically on 5–7 mm diameter paper disc. The fungal discs are then put in the center of the agar plate. The plates are then incubated at 25 ± 2 °C for 27 h [20] up to seven days. The colony diameter is measured in millimeters [215], while the toxicity of the extract is calculated in terms of the percentage of mycelial growth inhibition comparing the colony diameter of the poisoned plate (with bryophyte extract) and nonpoisoned plate (with distilled water) and calculated using the formula of Pandey et al., 1982 [216] C−T / C × 100, where C = average diameter (mm) of the fungal colony in the control plate and T = average diameter (mm) of fungal colony in treatment plate.

Disc diffusion assay [217]: Petri dishes are filled with Sabouraud dextrose agar and are seeded using a sterile cotton swab with 100 mL of fungal spore suspension diluted usually until 1–2 × 10^5^ spores/mL. A sterile filter disc of 6 mm diameter was placed into the plates and impregnated with bryophyte extract (0.5–10 mg/mL). Flucanazole (1–10 mg/mL), ketoconazole (50 µg/mL) or nystatin (10 µg/mL) are commonly used as a positive control in place of the extract. The plates are then incubated at 25 °C for 24/48 h. After incubation, the zones of growth inhibition around the discs are measured in millimeters [19].

Agar well diffusion assay: Petri dishes are filled with Sabouraud dextrose agar and seeded using a sterile cotton swab with 100 µL of fungal spore suspension usually diluted until 1–2 × 10^5^ spores/mL. A well of 6 mm diameter is cut into the center of plates and filled with up to 100 µL bryophyte extract (from 10 to 100 mg/mL). Fluconazole (1–10 mg/mL), ketoconazole (50 µg/mL) or nystatin (10 µg/mL) are used as a positive control. The plates are then incubated at 28 °C for 72 h. After incubation, the zone of growth inhibition around the well is measured in millimeters [23,218].

Autobiochromatographic TLC screening or direct bioautography: the plant extract diluted with the appropriate solvent is applied on the silica gel-coated plate. The plate is developed with a different ratio of solvents, e.g., ether [219] or acetone [15] to separate the phytochemicals. When dried, the plate is sprayed with a conidial suspension of 10^6^ spores/mL [220] and incubated for 48 h at 25 °C under humid condition. The fungal growth is evaluated under UV light [219]. In alternative, plates could be sprayed with tetrazolium salts (e.g., MTT), reincubated at 25 °C for 24 h or at 37 °C for 3–4 h [15,221]: clear, white zones against a purple background on the plate, due to the reduction of tetrazolium salts to formazan by the dehydrogenases of living fungi, indicate the antimicrobial activity of the bryophyte extract.

Microdilution method: the microdilution technique is performed in sterile 96-wells plate using a final volume of 200 μL. Inoculum of fungal spores is used at a final concentration of 1.0 × 10^6^ spores/mL in a volume of 100 μL per well. The bryophyte extracts are added to the broth medium with fungal inoculum to achieve the desired concentrations (0.05–20 mg/mL). The solvent used for preparing bryophyte extracts and a commercial fungicide (usually bifonazole at concentrations 0.1, 0.5 and 1 mg/mL) are used as a control. The plates are incubated for 72 h at 28 °C, and the minimum inhibitory concentration (MICs) of plant extracts is determined qualitatively at the binocular microscope by observing the presence of fungal growth, or quantitatively by determining the absorbance of each well at 620 nm with a microplate reader [222]. Moreover, several colorimetric methods based on the use of tetrazolium salts are often used for the MIC endpoint determination, in the antifungal microdilution method [223]. A variation of microdilution assay has been applied for the evaluation of antifungal potential of two mosses against *Aspergillus flavus* and *Candida albicans* [210].

Flow cytofluorometry: this technique is based on the use of a fluorescent dye, mainly propidium iodide (PI) that is an intercalating agent, for the detection of cell membrane damage. PI fluorescence intensity data are collected for 10,000 cells, using a single laser emitting excitation light at 488 nm, and cell membrane damage is demonstrated by an increase in PI staining. This parameter is displayed as the log of the mean PI fluorescence intensity on the x axis of a histogram chart. The flow cytofluorometric method is capable to discriminate three distinct subpopulations (dead, viable and injured cells), allowing one to evaluate the antifungal activity of the plant extract on the tested microorganism. Fungal spores suspension is mixed with an equal volume of plant extract and shook for the desired time, usually 25 °C for 24 h [224] or 10 min at room temperature [225]. Then, the cells are pelleted, resuspended in PI solution (25 µg/mL in phosphate-buffered saline) and incubated in the dark for 30 min at 32 °C. This method has been demonstrated to rapidly provide reproducible results, usually in 2–6 h.

### 5.2. In Vivo Methods

Mouse assay: to determine in vivo antifungal activity of bryophyte extracts, groups of male mice were challenged with 10^5^ fungal spores per mouse and later treated with different doses of plant extract (25, 50 and 100 mg/kg) or ketoconazole (as antifungal medication). One spore-challenged group, served as a control, received the solvent used for the bryophyte extract. Mortality of mice was observed daily for 30 days [226].

Plant protection assay: this method was applied in the pathosystem *Botrytis cinerea*/green pepper. Plants were sprayed with the bryophyte extracts (1% *m/v* in distilled water containing 0.0125% Tween20 as a surfactant) or with a fungicide as the control; after 4 h, the pathogen was inoculated by spraying a conidial suspension of 1 × 10^6^ conidia/mL. Plants are then incubated at 25 °C for 48 h. Disease severity was visually estimated as a percentage of infected leaf area in relation to total healthy tissues of sampled leaves [227].

## 6. Conclusions

Humankind has always been interested in plants not only as a source of food, but also as a tool for healing purposes; hence, since the herbal medicine represented the first and foremost therapeutic tool available to humans for many centuries, the history of plant-derived drugs—starting from Dioscoride’s *De materia medica*—is as old as the world. Currently, great resources are invested by multinational companies on herbal drugs and preparations. However, dragged by the timeless challenge between pathogens and their hosts, the global effort for the individuation and production of effective formulations has to contend between the insurgence of microbial resistance phenomena and an increasing necessity for environmental safety and sustainability.

Plants should be probably thought as the most formidable chemical laboratory that can be exploited for the production of an incredible number of biologically active molecules, and also bring the considerable advantage to perform as a renewable natural resource for these bioactives. The remarkable structural and chemical diversity possessed by such natural compounds cannot be matched by any synthetic libraries of small molecules, since they are evolutionarily optimized as drug-like molecules and remain the best sources of drugs and drug leads. In fact, this diversity is the result of the coevolution of each plant species with an even greater number of pathogens, predators and competitors: thus, unlike newly synthesized compounds, plant secondary metabolites are virtually guaranteed to exert biological activity that is highly likely to function in protecting the producing organism from, for example, a specific microbial pathogen.

The importance of botanical pesticides, and fungicide in particular, is attributed to their efficacy, biodegradability, varied modes of action, low toxicity and availability of source materials; however, to date, they are mostly obtained from higher and flowering-plants, probably due to the availability of long-dated knowledge background, a wider range of organ/tissue-based chemical diversity and a greater possibility of cultivation for industrial purposes. Bryophytes, non-vascular plants that left the aquatic environment for colonizing terrestrial lands around the Ordovician, evolved key adaptation mechanisms to cope with both abiotic and biotic stresses, including microbial pathogens, and contain many unique chemical compounds with high biological and ecological relevance. Hence, despite being lesser considered pivotal producers for natural pesticides than higher plants, their potential as a source of antifungal bioactives—to be exploited against both human and plant pathogens—should be emphasized, and it is worthy of investigation.

We aimed at reporting, reviewing and highlighting the various metabolites produced by bryophytes that have been found to exert antifungal bioactivity—or that, even if suspected to possess a potential, it has to be confirmed still, the present work represents our effort to contextualize them in their evolutionary, metabolic and investigational framework. Omics technologies and approaches, and the panel of biological assays overviewed, played (in the past, as they can definitely continue to play in the future) a unique role in individuating and/or characterizing molecules with antifungal properties buried in the treasure chest of these stunning, early-diverged land plants. In fact, beside already acknowledged bryoactives, for those compounds for which antifungal properties were not demonstrated yet, the hypothesis that, due to their biochemical derivation, a similar biological activity could be suggested, deserves further investigations.
